# Mas Receptor Agonist AVE0991 increases surfactant protein expression under hyperoxic conditions in human lung epithelial cells

**Published:** 2020-11-17

**Authors:** Ranga Prasanth Thiruvenkataramani, Amal Abdul-Hafez, Ira Gewolb, Bruce Uhal

**Affiliations:** 1 Division of Neonatology, Department of Pediatrics & Human Development, Michigan State University, USA; 2 Department of Physiology, Michigan State University, USA

**Keywords:** hyperoxia, surfactant proteins, bronchopulmonary dysplasia, BPD, human alveolar epithelial cells, local tissue renin-angiotensin system

## Abstract

**Background:**

Hyperoxia in pre-term neonates is a known risk factor of bronchopulmonary dysplasia (BPD). Hyperoxia is known to cause oxidative stress, inflammatory changes that leads to surfactant deactivation, and decreased surfactant expression. The previous research has shown short term exposure to hyperoxia increases surfactant protein expression but decreased expression in long term exposure. Local tissue renin-angiotensin system (RAS) is associated with tissue injury and repair and it may play a role in BPD. Endogenous peptide angiotensin 1–7 acts on the MAS receptor. The activation of the MAS receptor was previously shown to have protective pulmonary responses. However, the effect of MAS receptor activation on surfactant proteins in hyperoxic conditions has not been tested.

**Objective:**

To determine the effects of hyperoxia with or without MAS receptor activation on Surfactant proteins.

**Methods:**

Human epithelial cell line A549 and human primary alveolar epithelial cells (AECs) were cultured to sub-confluence (60–75%) and treated with hyperoxia (95% oxygen) and normoxia (21% oxygen) for 72 hours with or without the MAS receptor agonist (AVE0991) in serum-free F-12 nutrient media. Cells were lysed and cell lysates were collected for western blot. The statistical analysis was done using Student-Newman-Keuls Multiple comparison test.

**Results:**

Surfactant protein concentration increased in AVE treated group under the hyperoxic condition when compared to the control group in both A549 cells and human primary AECs. Surfactant protein was in higher concentration in AVE0991 treated cells in both hyperoxic and normoxic conditions when compared to the non-treated control group.

**Conclusions:**

MAS receptor activation via AVE0991 causes an increase in Surfactant protein concentration in both hyperoxic and normoxic conditions. As per our experiments, hyperoxic conditions decrease the production of surfactant protein when compared to normoxic conditions. These results may reveal a novel potential drug for BPD treatment and decrease its severity.

## Introduction

Prolonged increased oxygen exposure (hyperoxia) is a major determinant of Bronchopulmonary dysplasia (BPD) in preterm neonates. BPD is a chronic lung condition that has long-term morbidity in very low birth weight (VLBW) preterm neonates.^1,2^ BPD is associated with long-term morbidities like Neurodevelopmental deficits, exercise intolerance, reactive airway disease, and pulmonary hypertension, etc.,^3,4^ In 1967, Northway et al. first coined the term BPD and defined as sequelae of hyaline membrane disease/respiratory distress syndrome in preterm infants secondary to prolonged oxygen exposure and positive pressure ventilation.^5^ Lower the gestational age higher the incidence of BPD. Based on the NICHD consensus definition for BPD,^6^ the main criteria for BPD is the treatment of the neonate with oxygen of greater than 21% for at least 28 days of life. BPD severity can range from mild to severe based on the amount of oxygen required at 36 weeks corrected gestational age. With increasing survivability of VLBW preterm neonates, the incidence of Bronchopulmonary dysplasia (BPD) has been around 42%.^7^ The pathogenicity of BPD is multifactorial. Oxygen toxicity (hyperoxia), ventilator-associated injury, maternal infection (chorioamnionitis), antenatal steroids, immature immune system, genetics, and inflammation have all been implicated in the pathogenicity of BPD.^8^ In the last 3 decades, the histology of BPD has changed from predominant inflammation and fibrosis (*“OLD BPD”*) to alveolar developmental arrest (decreased septation, alveolar hypoplasia, and enlargement of existing alveoli- ‘Alveolar Simplification’), dysregulation of pulmonary vascular development (leading to pulmonary vascular disease like pulmonary hypertension) (*“NEW BPD”*).^8^ Oxygen induced acute lung injury causes an influx of inflammatory cells, increased pulmonary permeability leading to alveolar and interstitial fluid overload, oxidative stress, and epithelial cell injury/death.^10–12^ Hyperoxia leading to oxidative stress produces reactive oxygen species and that leads to the deactivation of surfactant proteins via a change in protein configuration.^13–15^ Hyperoxia causes alveolar epithelial cells apoptosis that leads to loss of epithelial barrier function which causes increased pulmonary permeability resulting in increased alveolar and interstitial edema, that in-turn leading to alveolar over flooding and washing out pulmonary surfactant.^16–19^ The lung edema is also a proteinaceous fluid leading to surfactant deactivation.^20^ Previous research has shown the variable expression of surfactant proteins in response to hyperoxia. Certain papers have suggested an increase in surfactant proteins secondary to hyperoxia exposure and some have suggested a decrease in surfactant protein with prolonged exposure to hyperoxia.^21–27^

**Figure 1:**
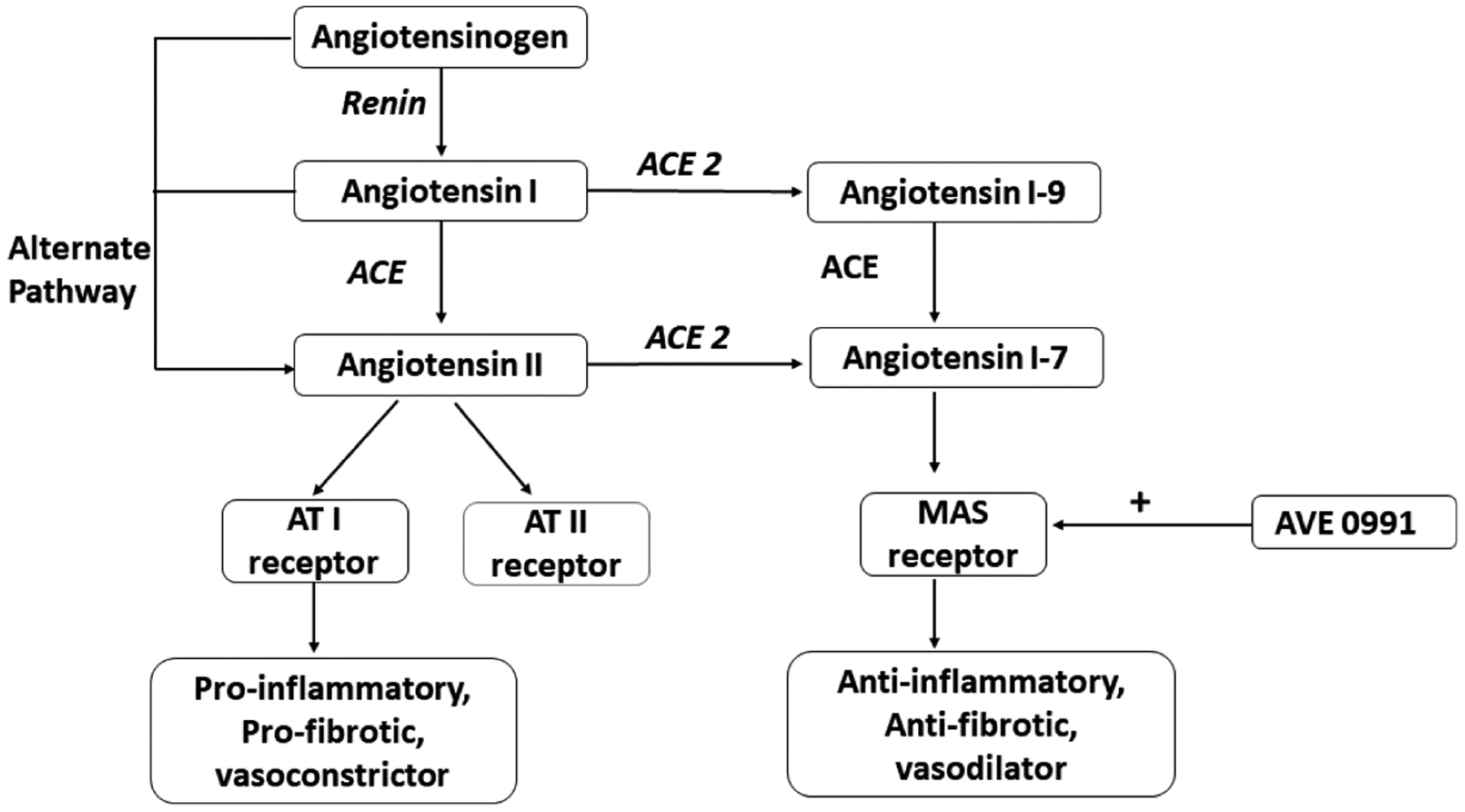
Local Tissue Renin-Angiotensin System.

Traditionally the renin-angiotensin system (RAS) is associated with blood pressure regulation,^28,29^ but the local tissue effect of RAS has been identified in heart, kidney, lungs, brain, pancreas, and adipose tissue.^44^ Several studies have shown that an imbalance in the levels of extracellular Angiotensin II and Angiotensin1-7 contributes to the pathogenesis of lung injury and defects in other organs.^50^ Local tissue activation of the RAS system through tissue injury may play a role in the pathogenesis of BPD and surfactant expression and function. In RAS, angiotensinogen is converted to angiotensin (Ang-I) by renin released from the kidney. As depicted in Figure 1, Angiotensin is converted to Angiotensin II (Ang-II, an octapeptide) by angiotensin-converting enzyme (ACE). Angiotensin1-7 (Ang-(1–7), a heptapeptide) is generated directly via the conversion of Ang-II by ACE-2 or indirectly via Ang-I by ACE-2 and ACE.^28^ Binding of Ang-II to Angiotensin type-1 receptor (AT-I) leads to vasoconstriction, proliferation, and fibrosis in multiple tissues, whereas binding of Ang-(1–7) to the MAS receptor induces vasodilation, inhibiting fibrosis, thrombogenesis, hypertension, cardiac hypertrophy and lung fibrosis.^28,30–33^ The balance between the opposing effector molecules Ang-II and Ang-(1–7) may play a pivotal role in tissue injury and recovery. The local RAS system is activated after tissue injury to promote tissue repair via the AT-II receptor, but abnormal activation via the AT-I receptor will lead to fibrosis.^33,34^ Previous research has shown that the angiotensin II (Ang-II) acting via the AT-I receptor leads to alveolar epithelial injury and apoptosis.^35–38^ Radioligand binding studies have provided evidence that Ang-(1–7) binds to the MAS receptor which is distinct from the AT-I and the AT-II receptor subtypes.^30^ Studies have shown Ang-(1–7) has a protective role on pulmonary cells via its action on the MAS receptor.^39–40^ AVE0991 (MAS receptor agonist) is a synthetic non-peptide analog of Ang-(1–7) which acts on the MAS receptor,^41^ exhibits a protective role on pulmonary cells, and shown to attenuate lung injury.^42–43^

Surfactant is primarily secreted from type-II alveolar epithelial cells and is important is decreasing the surface tension along the alveolar surface and thus preventing the collapse of the alveolus and improving gas exchange. Surfactant is important for lung function. Surfactant is mainly composed of phospholipids. The composition of surfactant is phosphatidylcholine desaturated-50%, phosphatidylcholine monosaturated (PC)-20%, phosphatidylglycerol (PG)– 8%, neutral Lipids–8%, other Phospholipids-6%, and surfactant proteins (A, B, C, D)–8%. SP-A and SP-D are hydrophilic surfactant proteins and SP-B and SP-C are hydrophobic surfactant proteins. The function of SP-A is to assists with tubular myelin formation (with SP-B and Calcium), enhances phospholipid uptake and inhibits secretion, host defense and SP-D is host defense. SP-B is critical for surfactant function, assists with tubular myelin formation (along with SP-A and Calcium), and promotes surface adsorption of Phospholipids (with SP-C). SP-C is critical for surfactant function, promotes Surface adsorption of Phospholipids (with SP-B).^9^

In this study, we investigate the changes in surfactant protein expression in response to hyperoxia. We also evaluate the effect of AVE0991 (MAS receptor activator) on surfactant protein expression under hyperoxic conditions.

## Methods

### Reagents and materials

The MAS receptor agonist AVE0991 was purchased from MedChem Express.

Penicillin streptomycin was purchased from Gibco (ref: 15140-122, Thermo fisher, USA), 0.25 % Trypsin- EDTA was purchased from Gibco (ref: 25200-056,Thermo fisher, USA), 6 well plates were purchased from CellPro, premium labware.

### Cell culture and treatment

The human lung adenocarcinoma A549 cell line was obtained from the American Type Cell Culture Collection (ATCC) and cultured in Ham’s F12 medium (ATCC, Manassas, VA), supplemented with 10% fetal bovine serum (FBS) (Gibco, Grand Island, NY), as described earlier. The cells were grown, maintained, and handled according to the supplier’s manual. All experiments were conducted in a serum-free Ham’s F12 medium. The cells were grown to 60–75 % sub-confluence in FBS supplemented medium and the medium was replaced with serum-free F12 medium and was cultured for 72 hours in either 21%O2, 5%CO2 (Normoxia) or 95%O2, 5%CO2 (Hyperoxia) in the presence or absence of 10^−7^M AVE0991. DMSO solvent was added in control groups for the absence of AVE0991. After 72 hours, cells were lysed with NP40. The lysates were centrifuged, and BCA protein analysis was performed. The lysates were used for surfactant protein detection.

Human primary alveolar epithelial cells (AECs) comprised of alveolar type1 and 2 epithelial cells were purchased from Sciencell research laboratories (Cat #3200). These cells were grown in Alveolar epithelial cell medium (Cat #3201), 10ml of fetal bovine serum (FBC) (Cat #0010), Epithelial cell growth supplement (EpiCGS, Cat #4152), penicillin/streptomycin solution (P/S cat#0503). The cells were grown in complete medium and then the experiment was done in a serum-free medium for 48 hours in either 21%O2, 5%CO2 (Normoxia), or in 95%O2, 5%CO2 (Hyperoxia) in the presence or absence of 10^−7^M AVE0991. DMSO solvent was added in control groups for the absence of AVE0991. After 48 hours, cells were lysed with NP40. The lysates were centrifuged, and BCA protein analysis was performed. The lysates were used for surfactant protein detection.

### Treatment of A549 cells with Actinomycin-D for transcription block

Actinomycin-D (A9415–5, 5 mg) was purchased from Sigma life science.

The A 549 cells were grown to 60–75% sub-confluence and the cells were treated with Actinomycin-D (0.2 −2ug/ml) for 72 hours. After 72 hours of incubation, cell lysates were used for surfactant protein detection.

### Western blotting

After performing a protein assay using the BCA method, 15–45 μg of protein lysate was loaded in each well of 4–20% Tris HCL polyacrylamide gels, and separated by SDS-PAGE, in 10× Tris/glycine/SDS buffer. Proteins were transferred to Polyvinylidene Difluoride “PVDF” blotting membrane and blocked by 5% nonfat dry milk in 0.1% tween 20 in Tris-buffered saline. Western blot analysis was performed using a polyclonal antibody against SP-C (SP-C (FL-197): sc-13979, Santa Cruz biotechnology inc, CA), a polyclonal antibody against SP- B (Genetex, Cat No: GTX55891), and a polyclonal antibody against SP-A (AB3420–1, EMD Millipore Crp., USA). PVDF membrane was incubated with an antibody for 16 h at + 4 °C. β-actin (Cell Signaling Technology, Danvers, MA) was used to normalize the assay. Bands were visualized by HRP-conjugated goat anti-rabbit antibody using enhanced chemiluminescence labelling (Amersham ECL Detection Reagent, GE Healthcare), we captured images by standard film techniques.

### Statistics

All data are shown as mean ± SEM. Group comparisons were evaluated by one-way analysis of variance (ANOVA). When the overall ANOVA was significant, comparisons between the groups were made using Student–Newman–Keuls post hoc test. For comparisons involving two groups, Student’s t-test was used. P < 0.05 was considered significant.

## Results

### MAS receptor activation prevents hyperoxia-induced decrease of SP-C in A549 cells

**Figure 2:**
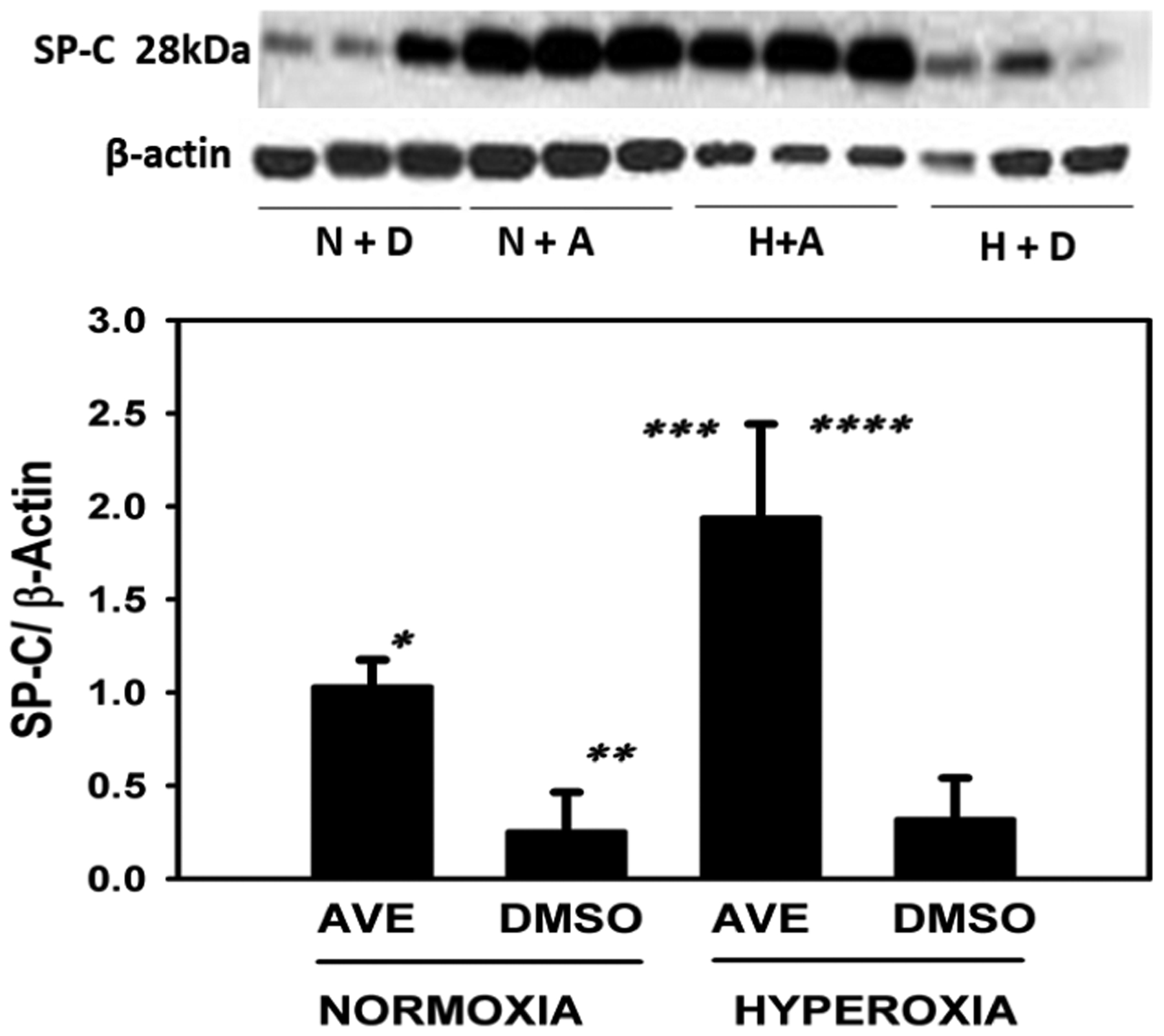
The MAS receptor agonist AVE-0991 restores and increases the SP-C in cultured alveolar epithelial cell (A549) monolayers under hyperoxic conditions (95% oxygen). A549 cells were cultured on 6 well plates to sub-confluence as described in Methods and were then exposed to 95% O2 in the presence or absence of MAS receptor agonist AVE0991. See Methods for details. Bars are the mean +/− S.E.M; * p<0.01 AVE Vs DMSO normoxia group, ** p<0.001 hyperoxia AVE vs normoxia DMSO group, *** p<0.01 AVE hyperoxia Vs AVE normoxia, **** p<0.001 AVE hyperoxia Vs DMSO hyperoxia using ANOVA Student-Newman-Keuls test, n=6. Western blot with control group and experimental group is shown and SP-C bands were noted at 28kDa and -actin was used for normalization.

SP-C was in higher concentration in AVE0991 treated cells in both hyperoxic and normoxic conditions when compared to the non-treated control group see figure 2. SP-C concentrations in AVE0991 treated cells were statistically higher (p<0.001) when compared to the non-treated control group. Under the hyperoxic conditions, SP-C concentrations in AVE0991 treated cells were statistically higher (3-fold increase, p<0.001) when compared to the non-treated control group. SP-C concentrations were statistically higher (p<0.01) between hyperoxic and normoxic conditions in cells treated with AVE0991. SP-C concentrations were not statistically different between hyperoxic and normoxic nontreated control groups.

### MAS receptor activation prevents hyperoxia-induced decrease of SP-B in A549 cells

**Figure 3:**
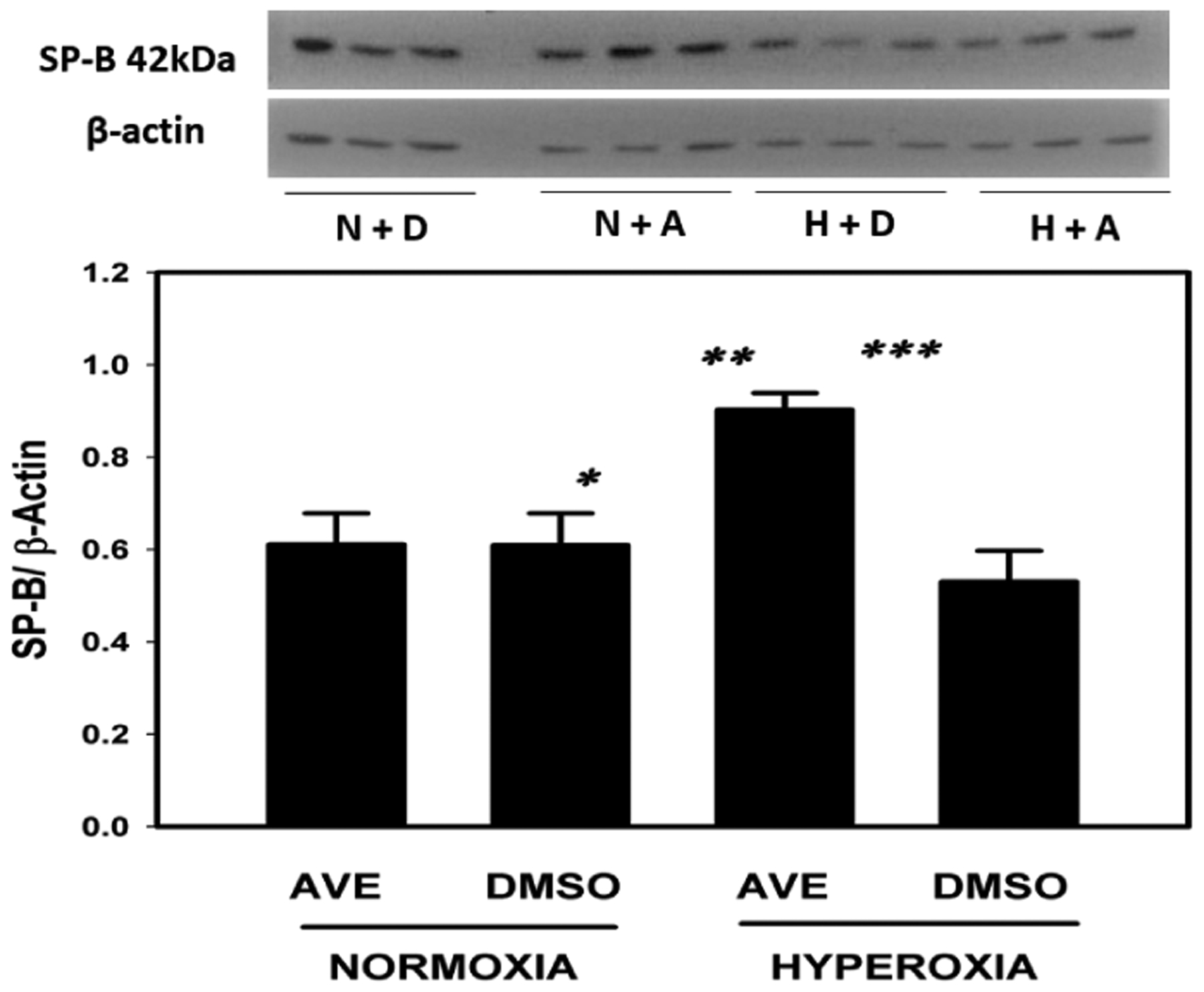
The MAS receptor agonist AVE-0991 restores and increases the SP-B in cultured alveolar epithelial cell A549 monolayers under hyperoxic conditions (95% oxygen). A549 cells were cultured on 6 well plates to sub-confluence as described in Methods and were then exposed to 95% O2 in the presence or absence of the MAS receptor agonist AVE0991. See Methods for details. Bars are the mean +/− S.E.M.; * p<0.01 AVE hyperoxia Vs DMSO normoxia, ** p<0.01 AVE hyperoxia vs AVE normoxia, *** p<0.001 AVE hyperoxia vs DMSO hyperoxia using ANOVA Student-Newman-Keuls test, n=6. Western blot with control group and experimental group is shown and SP-B bands were noted at 42kDa and -actin was used for normalization.

SP-B was in higher concentration in AVE0991 treated cells in hyperoxic conditions when compared to the non-treated control group see figure 3. SP-B concentration in AVE0991 treated cells under hyperoxic conditions was statistically higher (p<0.001) in the treated group when compared to the non-treated control group. SP-B concentrations were statistically higher (p<0.01) in hyperoxic group when compared to normoxic conditions in cells treated with AVE0991. SP-B concentrations were not statistically different between hyperoxic and normoxic non-treated control groups.

### MAS receptor activation prevents hyperoxia-induced decrease of SP-A in A549 cells

**Figure 4:**
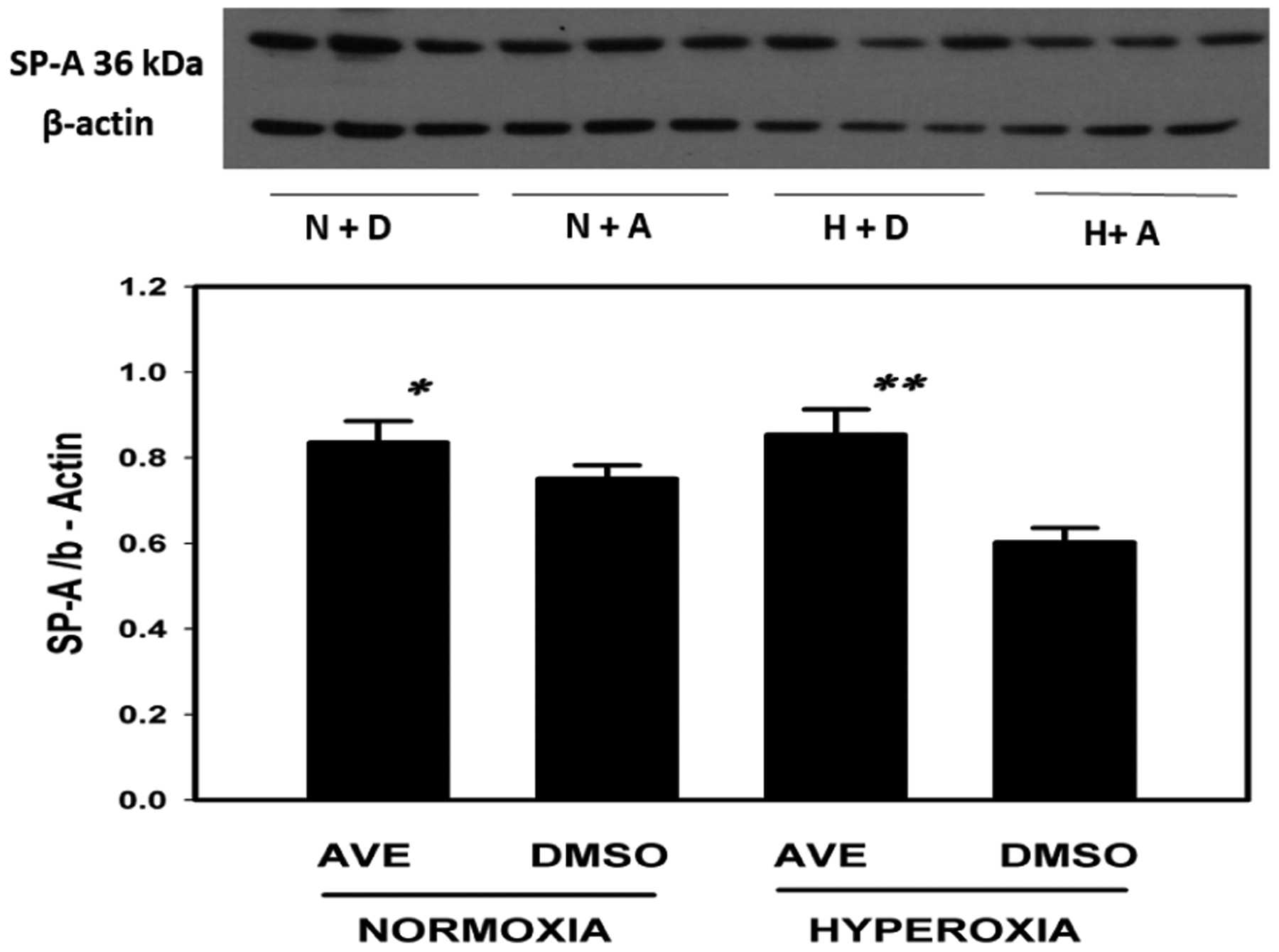
The MAS receptor agonist AVE-0991 restores and increases the SP-A in cultured alveolar epithelial cell monolayers under hyperoxic conditions (95% oxygen). A549 cells were cultured on 6 well plates to sub-confluence as described in Methods and were then exposed to 95% O2 in the presence or absence of the MAS receptor agonist AVE0991. See Methods for details. Bars are the mean +/− S.E.M.; * p<0.01 AVE normoxia vs DMSO hyperoxia, ** p<0.01 AVE hyperoxia Vs DMSO hyperoxia using ANOVA Student-Newman-Keuls test, n=6. Western blot with control group and experimental group is shown and SP-A bands were noted at 36kDa and -actin was used for normalization.

SP-A was in higher concentration in AVE0991 treated cells in both hyperoxic and normoxic conditions when compared to the non-treated control group see figure 4. SP-A concentrations in AVE0991 treated cells were statistically higher (p <0.01) in the hyperoxic treated group when compared to the non-treated control group. SP-A concentrations were not statistically different (P>0.05) between hyperoxic and normoxic conditions in cells treated with AVE0991. SP-A concentrations were not statistically different between hyperoxic and normoxic non-treated control groups.

### MAS receptor activation increases SP-C under the hyperoxic condition in Primary human AECs

**Figure 5:**
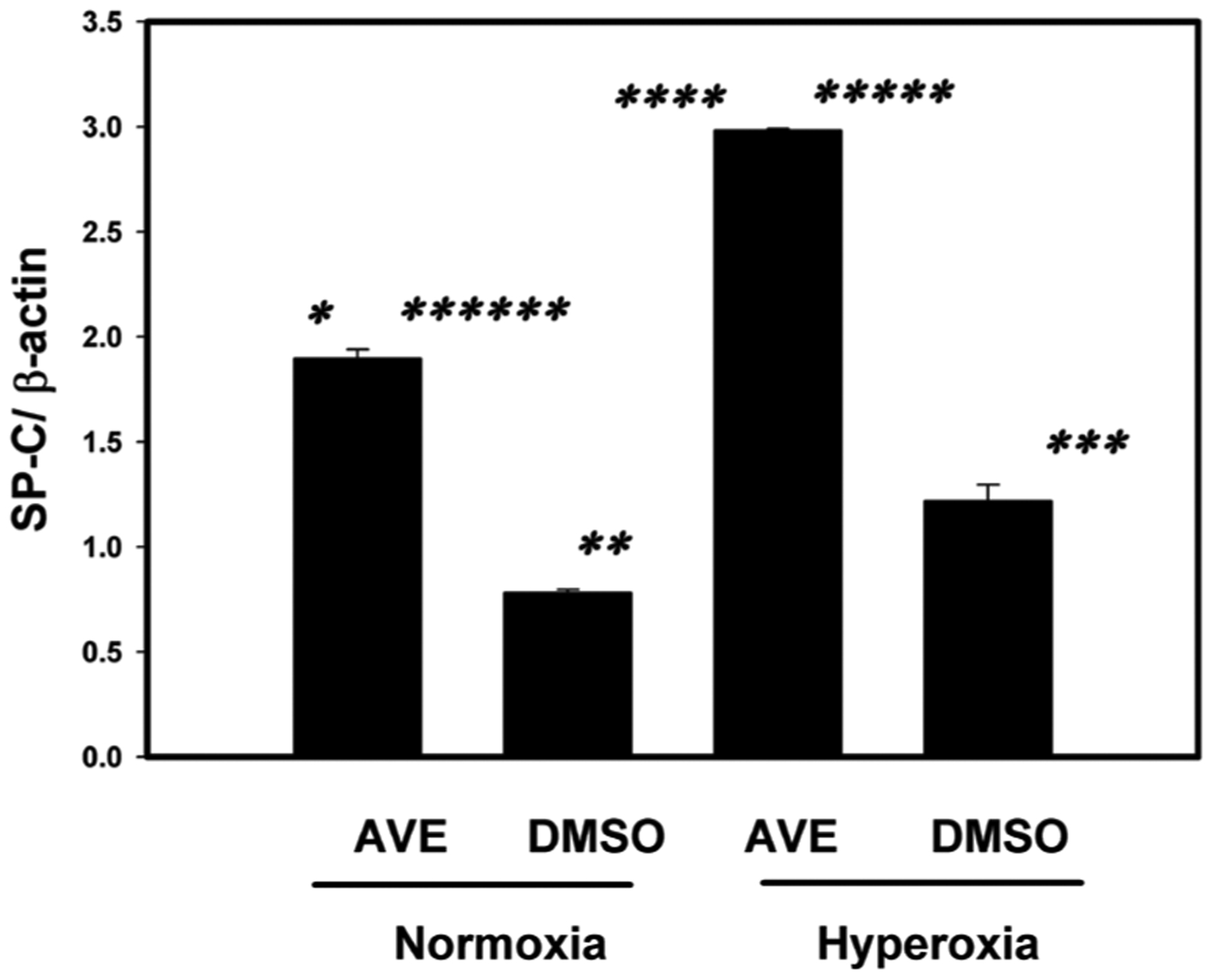
The MAS receptor agonist AVE-0991 restores and increases the SP-C in cultured human primary AECs monolayers under hyperoxic conditions (95% oxygen). Human primary AECs were cultured on 6 well plates to sub-confluence as described in Methods and were then exposed to 95% O2 in the presence or absence of the MAS receptor agonist AVE0991. See Methods for details. Bars are the mean +/− S.E.M.; * p<0.001 AVE normoxia Vs DMSO normoxia, ** p<0.001 AVE hyperoxia vs DMSO normoxia, *** p<0.001 DMSO hyperoxia vs DMSO normoxia, **** p<0.001 AVE hyperoxia vs AVE normoxia, ***** p<0.001 AVE hyperoxia vs DMSO hyperoxia, ****** p<0.001 DMSO hyperoxia vs AVE normoxia using ANOVA Student-Newman-Keuls test, n=3.

SP-C was in higher concentration in AVE0991 treated cells in both hyperoxic and normoxic conditions when compared to the nontreated control group see figure 5. SP-C concentrations in AVE0991 treated cells were statistically higher (p<0.001) when compared to the non-treated control group in both normoxic and hyperoxic group. SP-C concentrations in AVE0991 treated cells under hyperoxic conditions were statistically higher (p<0.001) when compared to the nontreated control group in both hyperoxic (3-fold increase) and normoxic group (5-fold increase) and also higher than AVE0991 treated normoxic group. SP-C concentration was statistically higher in the hyperoxic group when compared to the normoxic group in the non-treated control group. SP-C concentration was also higher in the AVE0991 treated normoxic group when compared to the control normoxic group.

### MAS receptor activation increases SP-B under the hyperoxic condition in Primary human AECs

**Figure 6:**
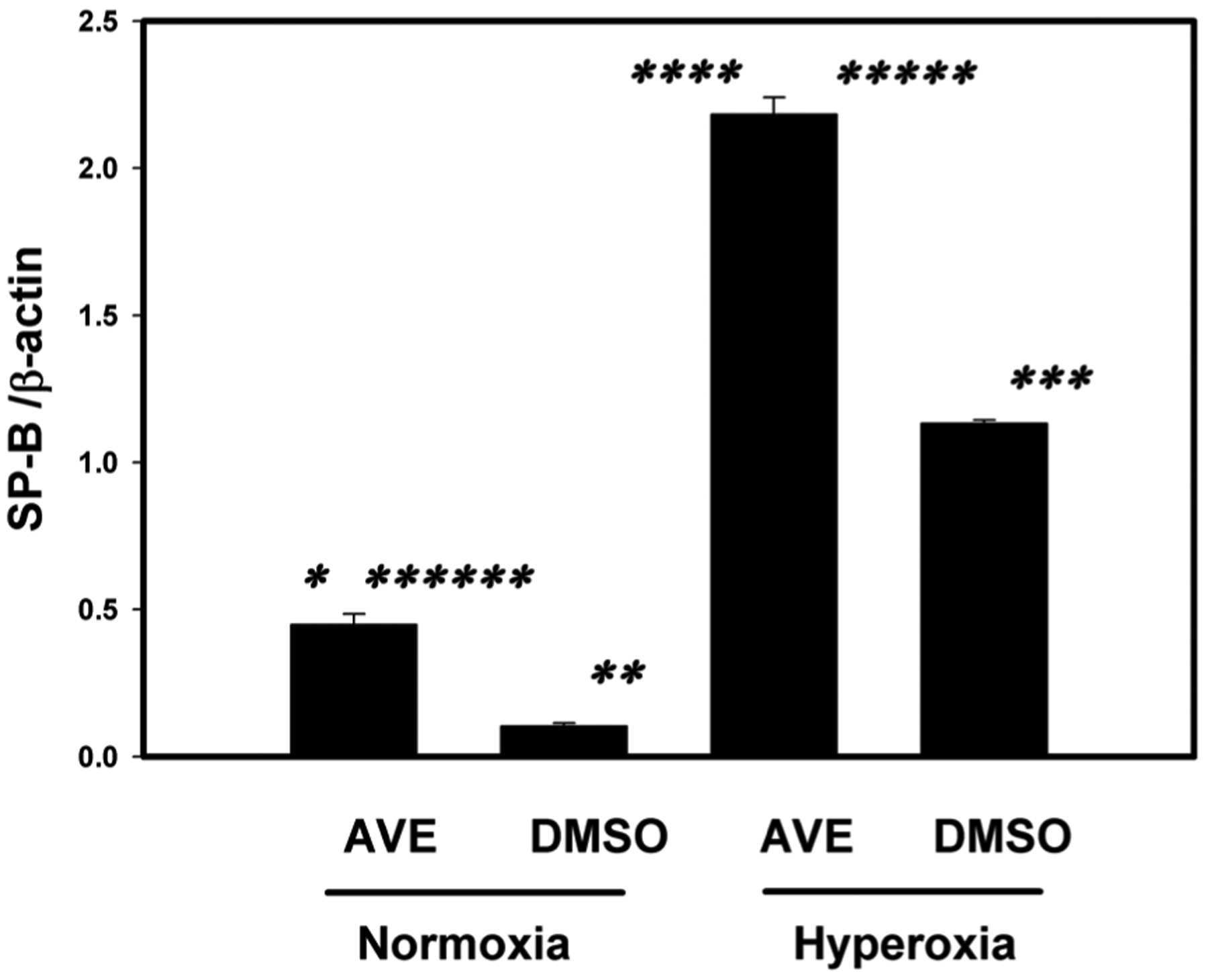
The MAS receptor agonist AVE-0991 restores and increases the SP-B in cultured human primary AECs monolayers under hyperoxic conditions (95% oxygen). Human primary alveolar AECs were cultured on 6 well plates to sub-confluence as described in Methods and were then exposed to 95% O2 in the presence or absence of the MAS receptor agonist AVE0991. See Methods for details. Bars are the mean +/− S.E.M.; * p<0.001 AVE normoxia Vs DMSO normoxia, ** p<0.001 AVE hyperoxia vs DMSO normoxia, *** p<0.001 DMSO hyperoxia vs DMSO normoxia, **** p<0.001 AVE hyperoxia vs AVE normoxia, ***** p<0.001 AVE hyperoxia vs DMSO hyperoxia, ****** p<0.001 DMSO hyperoxia vs AVE normoxia using ANOVA Student-Newman-Keuls test, n=3.

SP-B was in higher concentration in AVE0991 treated cells in both hyperoxic and normoxic conditions when compared to the nontreated control group see figure 6. SP-B concentrations in AVE0991 treated cells were statistically higher (p<0.001) when compared to the non-treated control group in both normoxic and hyperoxic group. SP-B concentrations in AVE0991 treated cells under hyperoxic conditions were statistically higher (p<0.001) when compared to the nontreated control group in both hyperoxic (2-fold increase) and normoxic group (4-fold increase) and also higher than AVE0991 treated normoxic group (4-fold increase). SP-B concentration was statistically higher in hyperoxic group (2-fold increase) when compared to the normoxic group in non-treated control group. SP-B concentration were also higher in AVE0991 treated normoxic group when compared to untreated normoxic group.

### MAS receptor activation increases SP-A under the hyperoxic condition in Primary human AECs

**Figure 7:**
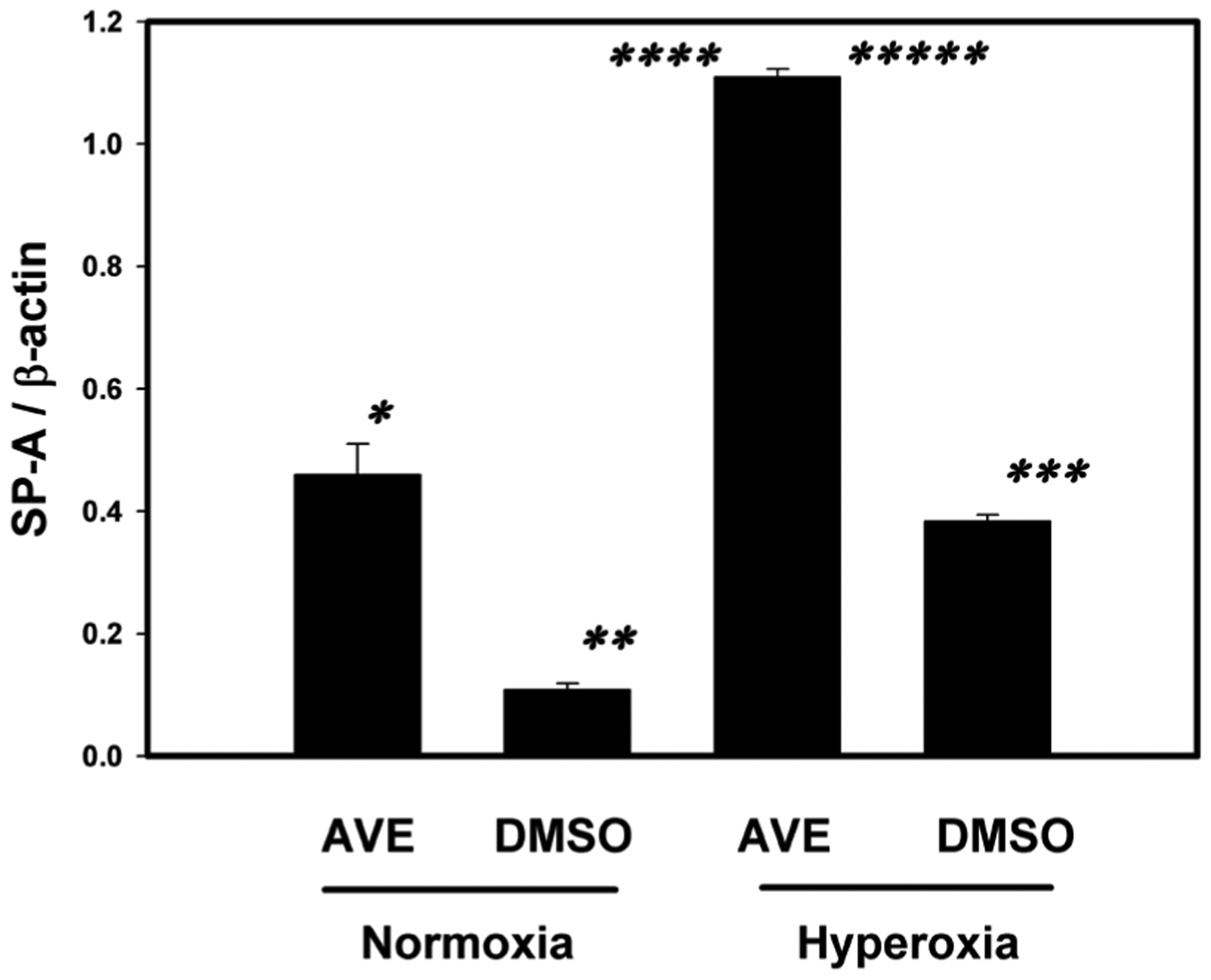
The MAS receptor agonist AVE-0991 restores and increases the SP-A in cultured human primary AECs monolayers under hyperoxic conditions (95% oxygen). Human primary AECs were cultured on 6 well plates to sub-confluence as described in Methods and were then exposed to 95% O2 in the presence or absence of the MAS receptor agonist AVE0991. See Methods for details. Bars are the mean +/− S.E.M.; * p<0.001 AVE normoxia Vs DMSO normoxia, ** p<0.001 AVE hyperoxia vs DMSO normoxia, *** p<0.001 DMSO hyperoxia vs DMSO normoxia, **** p<0.001 AVE hyperoxia vs AVE normoxia, ***** p<0.001 AVE hyperoxia vs DMSO hyperoxia using ANOVA Student-Newman-Keuls test, n=3.

SPA was in higher concentration in AVE0991 treated cells in both hyperoxic and normoxic conditions when compared to the nontreated control group see figure 7. SPA concentrations in AVE0991 treated cells were statistically higher (p<0.001) when compared to the non-treated control group in both normoxic and hyperoxic group. SP A concentrations in AVE0991 treated cells under hyperoxic conditions were statistically higher (p<0.001) when compared to the nontreated control group in both hyperoxic (3-fold increase) and normoxic group and also higher than AVE0991 treated normoxic group (2.5-fold increase). SP-A concentration was statistically higher (2-fold increase, P <0.001) in hyperoxic group when compared to normoxic group in non-treated control group. SP-A concentration were also higher in AVE0991 treated normoxic group when compared to untreated normoxic group.

## Discussion

Hyperoxic lung injury is a risk factor for bronchopulmonary dysplasia (BPD) in preterm babies. Hyperoxia causes inflammatory changes in the lungs,^10–12^ reactive oxygen species (ROS) formation and that in turn altering the structure of surfactant proteins and rendering them non-functional.^13–15^ Hyperoxia also causes apoptosis of Alveolar epithelial cells,^45,46^ and disruption of epithelial barrier leading to over flooding of alveolus causing surfactant wash-out,^16–19^ and proteinaceous lung edema causing surfactant deactivation,^20^ leading to surfactant deficiency in at-risk preterm neonates. There are multiple single center studies and currently ongoing clinical trials of surfactant administration for preterm babies for decreasing the severity of BPD. To our knowledge, the effect of AVE0991 on surfactant proteins under hyperoxic condition was never studied.

Wagenaar et al.^39^ showed administration of MAS receptor agonist decreased alveolar septal thickness, decreased influx of neutrophils, and decreased arterial medial wall thickness in the hyperoxic condition when compared to the normoxic condition in rat pups. He also demonstrated that there were no beneficial effects on hyperoxia-induced inhibition of alveolarization, angiogenesis, and an influx of macrophage. The data from this study suggests MAS receptor agonist may play a role in decreasing the inflammation secondary to hyperoxia-induced lung injury. In our lab, Gopallawa et al.^40^ have shown MAS receptor is present in A549 cells and inhibition of MAS receptor has shown to decrease the protective effect of Ang1-7 and increase in apoptosis.

Jin et al.^27^ demonstrated long-term exposure to hyperoxia inhibits the alveolar development and increased apoptosis of alveolar epithelial cells. It was also shown SP-C and SP-D content in the hyperoxia group was lower than that in the air group on the 1^st^ day, increased on the 3^rd^day, reached the peak on the 7^th^day, and began to decrease on the 10^th^ day, and more obvious decrease on the 14^th^ day. Previous work in our lab has demonstrated, hyperoxia causes increased apoptosis of alveolar epithelial cells and the MAS receptor agonist AVE0991 has led to the restoration of alveolar epithelial barrier integrity by decreasing AECs apoptosis.^47^ In our study, we demonstrated the decrease in surfactant proteins expression (SP-A, SP-B, and SP-C) in hyperoxia exposed group when compared to normoxia group in A549 cells, but in human primary AECs surfactant protein expression was higher in the untreated hyperoxia group when compared to the untreated normoxic group. In human primary AECs, under hyperoxic conditions, like previous studies, there was an increased expression of surfactant protein after short term exposure secondary to a stress response. Activation of the MAS receptor via AVE0991 causes an increase in or restoration of surfactant protein (SP-A, SP-B and SP-C) in the hyperoxic group when compared to the control group in both A549 cells and human primary AECs. Also, activation of the MAS receptor via AVE0991 causes an increase in surfactant protein in the treatment group (both in hyperoxic and normoxic group) when compared to control group in A549 cells. In human primary AECs results were similar to A549 cells demonstrating activation of the MAS receptor with AVE0991 causes a robust increase (multiple folds) in surfactant protein (SP-A, SP-B, and SP-C) in hyperoxia exposed treated group when compared to control group.

In our study, an increase in SP-C under treatment group with AVE0991 under hyperoxic condition is robust when compared to SP-B and SP-A in A549 cells. One possible explanation is SP-C is the only surfactant protein expressed by type-II alveolar epithelial cells whereas SP-B and SP-A are expressed by type-II alveolar epithelial cells and also in other cells in the respiratory tract and Clara cells. Also, the increase in surfactant protein in A459 cells can be explained by the propensity of A549 cells differentiation towards type-2 pneumocyte and also treatment with MAS agonist AVE0991 may decrease the epithelial-mesenchymal transitioning in A549 cells and that in turn lead to increased expression or restoration of surfactant protein expression in hyperoxia treated cells. In our lab, it is also shown, the MAS receptor activation by AVE0991 has led to decreased apoptosis in hyperoxia treated A549 cells.^47^ The MAS receptor activation increased surfactant proteins by decreasing apoptosis secondary to hyperoxia is also another possible explanation. In primary human AECs, the hyperoxia exposure was decreased to 48 hours Vs 72 hours in A549 cells because the propensity of the type-II cells to transition to type-I cells with prolonged incubation (Day4–10) has resulted with an increase in type-I cell marker and decrease in surfactant protein expression. The increase in surfactant proteins in primary human AECs by the MAS agonist AVE0991 suggests, AVE0991 may prevent transitioning of type-2 pneumocytes to type-1 pneumocytes leading to normal sustained surfactant expression or increase in total surfactant protein production in hyperoxia treated cells. The Mas receptor activation also causes decrease in apoptosis of the cells. The increase in hydrophobic surfactant proteins (SP-B and SP-C) may play an important role in decreasing the surface tension along the alveolar surface by creating a liquid-air interface and also the increase in hydrophilic surfactant protein (SP-A) help spread the surfactant evenly through the alveolar surface. This in turn may lead to better gas exchange, decrease oxygen requirement, and decrease inflammatory changes in lung and decrease the severity of BPD.

White et al. showed upregulation of surfactant proteins (SP-A, SP-B, and SP-C) at both the pre-translational and post-translational levels in distal lung epithelium during adaptation to hyperoxia in the newborn rat.^21^ But other studies have reported an increase in mRNA in AECs in response to hyperoxia but a varied expression of surfactant proteins.^22–24^ In the recently published study, it was shown prolonged exposure to Hyperoxia (> 60 hours) will lead to a decrease in surfactant proteins and increased apoptosis of alveolar epithelial cells.^25–26^ In our study, we used Actinomycin-D, a transcription inhibitor with and without AVE0991. In this experiment, Actinomycin-D was very toxic to cells leading to apoptosis of cells even under multiple decreased concentrations. Due to this, the role of MAS agonist AVE0991 in intracellular signaling was unestablished at the transcription level. The pulmonary protective effect of MAS receptor activation suggests more study is required to elucidate the mechanism of action.

In previous work in our lab, Uhal et al.^29^ has demonstrated in in-vitro studies, that the binding of Ang-II to its AT-I receptor causes phosphorylation of c-JUN-NH2-terminal kinase (JNK), a member of the mitogen protein kinase (MAPK) family, which is required for AEC apoptosis. Mitogen-activated protein kinase phosphatases (MKPs) belong to the family of dual-specificity phosphatases (DUSPs), which are important negative regulators of MKPs through dephosphorylating the Thr-X-Tyr motif of MKPs.^49^ Uhal et al.^48^ also demonstrated JNK phosphorylation is a required event in AEC apoptosis in response to the binding of Ang-II to the AT-I receptor. It was also showed that bleomycin- or Ang-II induced JNK phosphorylation and apoptosis were blocked by Ang-(1–7) through its binding to the MAS receptor. In a recent publication, it is shown the MAS receptor agonist AVE0991 restores hyperoxia-induced loss of lung epithelial barrier function through inhibition of apoptosis.^47^ At baseline (without stimulation) Ang-(1–7) is more abundant than Ang-II in the cell culture media bathing primary human AECs and that Ang-(1–7) dephosphorylates p-JNK as a cell survival mechanism.^48^ Gopallawa et al.^40^ showed that the ability of Ang-(1–7) to block Ang-II induced p-JNK, caspase-9, MMP, DNA fragmentation, and apoptosis is abolished if MKP-2 is silenced. Ang-(1–7) upregulates the phosphatase MKP-2 through the MAS receptor and thereby maintains low p-JNK levels to promote AEC survival by preventing apoptosis. Blockade or knockdown of the MAS receptor by the MAS receptor antagonist A779 or antisense oligonucleotides attenuated the induction of MKP-2 by ANG-(1–7) and confirmed that MAS acts through MKP-2. Collectively, these studies showed that the upregulation of MKP-2 by the ANG-(1–7)/MAS pathway constitutively dephosphorylates JNK and prevents apoptosis.

This study was primarily done on A549 cells which may not reflect neonatal alveolar epithelial cells is a limitation of the study. But we conducted similar experiments in human adult primary epithelial cells and the results from A549 cells were confirmed but it was an adult primary AECs, and this still may not apply to the neonatal lung.

In summary, our study suggests MAS receptor agonist AVE0991 or activation MAS receptor may yield a potential therapeutic agent in the treatment of at-risk preterm neonates to decrease the severity of BPD or neonates with established BPD.

## Abbreviations:

A549: human lung adenocarcinoma 549 cell line
ACE: angiotensin-converting enzyme
AECs: human primary alveolar epithelial cells
Ang: angiotensin
AT-I: angiotensin type-1 receptor
AT-II: angiotensin type-2 receptor
BPD: bronchopulmonary dysplasia
CLD: chronic lung disease
CO_2_: carbon dioxide
DMSO: dimethyl sulfoxide
HRP: horseradish peroxidase
O_2_: oxygen
RAS: renin-angiotensin system
SDS PAGE: sodium dodecyl sulphate–polyacrylamide gel electrophoresis
SEM: standard error of mean
SP [-A, -B, -C and – D]: surfactant protein [A, B, C and D respectively]
VLBW: very low birth weight preterm neonates
NICHD: national institute of child health and human development
